# Bone Marrow Stromal Cells Modulate Mouse ENT1 Activity and Protect Leukemia Cells from Cytarabine Induced Apoptosis

**DOI:** 10.1371/journal.pone.0037203

**Published:** 2012-05-22

**Authors:** Patricia Macanas-Pirard, Andrea Leisewitz, Richard Broekhuizen, Kelly Cautivo, Francisco M. Barriga, Francisco Leisewitz, Victoria Gidi, Erick Riquelme, Viviana P. Montecinos, Pilar Swett, Pelayo Besa, Pablo Ramirez, Mauricio Ocqueteau, Alexis M. Kalergis, Matthew Holt, Michael Rettig, John F. DiPersio, Bruno Nervi

**Affiliations:** 1 Departamento de Hematología y Oncología, Facultad de Medicina, Pontificia Universidad Católica de Chile, Santiago, Chile; 2 Departamento de Reumatología, Facultad de Medicina, Pontificia Universidad Católica de Chile, Santiago, Chile; 3 Departamento de Genética Molecular y Microbiología, Facultad de Ciencias Biológicas, Millennium Institute on Immunology and Immunotherapy, Pontificia Universidad Católica de Chile, Santiago, Chile; 4 Oncology Division, Washington University School of Medicine, Saint Louis, Missouri, United States of America; Emory University, United States of America

## Abstract

**Background:**

Despite a high response rate to chemotherapy, the majority of patients with acute myeloid leukemia (AML) are destined to relapse due to residual disease in the bone marrow (BM). The tumor microenvironment is increasingly being recognized as a critical factor in mediating cancer cell survival and drug resistance. In this study, we propose to identify mechanisms involved in the chemoprotection conferred by the BM stroma to leukemia cells.

**Methods:**

Using a leukemia mouse model and a human leukemia cell line, we studied the interaction of leukemia cells with the BM microenvironment. We evaluated in vivo and in vitro leukemia cell chemoprotection to different cytotoxic agents mediated by the BM stroma. Leukemia cell apoptosis was assessed by flow cytometry and western blotting. The activity of the equilibrative nucleoside transporter 1 (ENT1), responsible for cytarabine cell incorporation, was investigated by measuring transport and intracellular accumulation of ^3^H-adenosine.

**Results:**

Leukemia cell mobilization from the bone marrow into peripheral blood in vivo using a CXCR4 inhibitor induced chemo-sensitization of leukemia cells to cytarabine, which translated into a prolonged survival advantage in our mouse leukemia model. In vitro, the BM stromal cells secreted a soluble factor that mediated significant chemoprotection to leukemia cells from cytarabine induced apoptosis. Furthermore, the BM stromal cell supernatant induced a 50% reduction of the ENT1 activity in leukemia cells, reducing the incorporation of cytarabine. No protection was observed when radiation or other cytotoxic agents such as etoposide, cisplatin and 5-fluorouracil were used.

**Conclusion:**

The BM stroma secretes a soluble factor that significantly protects leukemia cells from cytarabine-induced apoptosis and blocks ENT1 activity. Strategies that modify the chemo-protective effects mediated by the BM microenvironment may enhance the benefit of conventional chemotherapy for patients with AML.

## Introduction

Acute myeloid leukemia (AML) is a clonal disorder of the hematopoietic stem cell (HSC) characterized by the accumulation of myeloblasts in the bone marrow (BM) and peripheral blood. Despite a high response rate to chemotherapy, the vast majority of patients with AML relapse due to residual disease in the BM. Current treatments based on chemotherapy alone may cure nearly 30–40% of patients less than 60 years old, and approximately 10% of patients over 60. [1].

The HSC resides in the BM and interacts with a highly organized microenvironment comprised of stromal cells, osteoblasts, osteoclasts and an extracellular matrix rich in fibronectin, collagens and various proteoglycans. [2], [3] These BM cells secrete different regulatory soluble signals, including cytokines, chemokines and growth factors that regulate trafficking as well as self-renewal, proliferation, and differentiation of HSC. Previous reports have shown that the BM niche confers protection to hematologic malignancies and serves as support for epithelial metastasis. [4], [5], [6] In fact, the interaction of leukemia cells with the BM stroma has been proposed as a mechanism for chemotherapy resistance. [4].

Cytarabine (Ara-C) is considered the single most effective agent for the induction of remission in the treatment of AML and is incorporated into virtually all standard chemotherapy regiments. [5] The antileukemic effect of Ara-C depends on metabolic activation and drug uptake. [6] Ara-C is a nucleoside analog that is phosphorylated into its active form cytosine arabinoside triphosphate (Ara-CTP), which competes with deoxycytidine triphosphate (dCTP) for incorporation into DNA. Ara-C blocks DNA synthesis thereby inducing cellular apoptosis. Nucleoside analogues like Ara-C are hydrophilic molecules and therefore require specialized membrane transport proteins to be transported into cells. [7] The uptake of Ara-C into cells is mediated primarily via the equilibrative nucleoside transporter 1 (ENT1). The ENTs are integral membrane proteins responsible for the uptake of a large number of nucleosides broadly used in cancer treatment such as Ara-C, gemcitabine, and fludarabine. [8], [9] The deficiency of hENT1 has been suggested as a mechanism of cellular resistance to Ara-C. [10], [11].

We have previously described a murine leukemia model that exhibits the characteristics of microenvironment-mediated drug resistance, and we showed that the interaction between leukemia cells and the stroma can be blocked in vivo by AMD3100, a small molecule inhibitor of CXCR4 that mobilizes normal hematopoietic stem cells and leukemic blasts from hematopoietic niches into the peripheral blood. [12] Disruption of the CXCR4/SDF-1 axis by AMD3100 improved overall survival of mice when treated with Ara-C. This effect may have been mediated through synergistic cytotoxicity, mitigation of stromal-cell derived chemoprotection, or enforced cell cycling with a loss in quiescence. To further understand the mechanisms involved in this chemoprotection conferred by the BM stroma, we developed in this study an in vitro co-culture system of a mouse BM derived stromal cell line and leukemia cells to determine the effect of stromal cells on leukemia cell apoptosis induced by cytotoxic agents. Interestingly, we observed that BM stromal cells provided a specific preferential protection for Ara-C induced apoptosis not observed with other cytotoxic agents. This protection was mediated by a soluble factor(s) produced by the stroma that also modulates mouse ENT1 activity.

## Materials and Methods

### In vivo Studies

#### Mice

C57BL/6J and 129Sv/J mice were obtained from the Jackson Laboratory (Bar Harbor, ME, USA). The mCG^PR/+^ strain has been previously described and was maintained on a C57BL/6 × 129/SvJ F1 background. [13] Hybrid C57BL/6J × 129Sv/JF1 (B6129F1) mice at 9 to 18 weeks of age were used in all the experiments. Animal care and euthanasia protocols were approved by the Bioethics and Biosafety Commission of the Faculty of Biological Sciences, Pontificia Universidad Católica de Chile (approval ID: CBB-2008).

#### Acute promyelocytic leukemia cells and transplantation

Acute promyelocytic leukemia cells (APL) from the spleens of mCG-PML-RAR knock in mice (B6129F1) were harvested and cryopreserved. [13] APL cells (10^6^ cells/mouse) were injected intravenously via the tail vein into genetically compatible B6129F1 recipients, without pretreatment with any radiation or chemotherapy conditioning.

#### Mobilization protocol and treatments

Plerixafor (AMD3100) (Genzyme, Cambridge, MA) was supplied as a sterile isotonic aqueous solution at 20 mg/ml and was administered at a dose of 5 mg/Kg as a subcutaneous injection 1 hour before and 3 hours after each Ara-C injection. Mice treated with chemotherapy received a single subcutaneous injection of Ara-C (500 mg/Kg) (Pfizer, Bentley, WA, Australia) on days 12 and 13 after APL injection. Mice treated with radiotherapy received 350 cGy on day 12 after APL injection or the combination.

#### Blood counts and flow cytometry

To ensure leukemia development and engraftment, peripheral blood samples were taken from the tail of mice for complete blood counts using an automated cell counter (Sysmex KX-21N, Sysmex America, Inc., Mundelein, IL) and flow cytometry (BD FACS Canto II, BD Biosciences, San Diego, CA). The spleens from dead or euthanized animals were analyzed for evidence of acute leukemia. Single-cell suspensions from blood samples were stained with fluorescein isothiocyanate (FITC)-conjugated anti-mouse CD117, phycoerythrin (PE)-conjugated anti-mouse CD34 and allophycocyanin (APC)-conjugated anti-mouse Ly-6G and Ly-6C (myeloid differentiation antigen, Gr-1) (all from BD Biosciences Pharmingen, San Diego, CA). A minimum of 10,000 events were acquired for each sample by FACS and data analyzed using FACSDiva software (BD Biosciences, San Diego, CA).

### In vitro Studies

#### Cell culture and reagents

The mouse bone marrow stromal cell line M2-10B4 (M2-BMSCs), the human BMSCs HS-5 (HS5-BMSCs) and the leukemia APL cells were kindly provided by Dr. John DiPersio (Washington University School of Medicine, St. Louis, USA). The U-937 AML cell line was purchased from ATCC (Manassas, VA). Primary mouse BMSCs were isolated as previously described. [14] All cell lines were cultured in RPMI 1640 (Invitrogen, Carlsbad, CA, USA), supplemented with 5% (v/v) FBS, 100 IU/ml penicillin and 100 µg/ml streptomycin, non-essential amino acids and 2 mM L-glutamine in a humidified incubator at 37°C with 5% carbon dioxide. To prepare supernatant from M2-10B4 or HS-5, cells were grown for 24 hours in supplemented RPMI to near confluence, centrifuged at 1,000 *g* for 5 minutes. Cell-free culture supernatants were obtained by passage through a 0.45 µm sterile filter. Nitrobenzylmercaptopurine (NBMPR) was obtained from Sigma Chemical Co. (St. Louis, MO). Human fibronectin was purchased from BD Biosciences, San Diego, CA. 24-well plates were coated with fibronectin according to the manufacturers instructions. Briefly, 1 ml of 9 µg/ml of fibronectin in sterile water was aliquoted per well and incubated at room temperature for 1 hour. Then, carefully removed supernatant and washed wells with sterile PBS. Plates were allowed to dry and stored at 4°C until used. Transwell plates with permeable supports and microporous membranes (0, 45 µm) were purchased from Corning, Pittsburgh, PA. 

#### Treatment of AML cells

AML cells cultured alone, or co-cultured with M2-BMSCs, M2-BMSCs supernatant (M2-BM SN) or primary mouse BM supernatant (PM-BM SN) were exposed to cytotoxic agents such as Ara-C (125, 250 and 500 ng/ml), gemcitabine (50, 100 and 200 ng/ml), epirubicin (3.8, 7.5 and 15 ng/ml), etoposide (150, 300 and 600 ng/ml), cisplatin (250, 500 and 1000 µg/ml) and 5-fluorouracil (100, 250 and 500 ng/ml) for 24 hours before cell viability analysis. All chemotherapeutic agents were purchased from Pfizer (Bentley, WA, Australia). APL cells cultured alone, or co-cultured with BMSCs were also exposed to radiotherapy (200, 400 and 600 cGy) with 24 hours cell recovery before analysis cell viability. Human U-937 cells were cultured with or without human HS5-BM supernatant (HS5-BM SN) and exposed to Ara-C for 24 hours (300 and 600 ng/ml) before analysis of cell viability.

#### Detection of cell viability

Human or murine AML cells were cultured in 96-well plates with or without BM SN for 2 hours before treatment with cytotoxic drugs for 24 hours. Cell viability was assessed by the MTT (3-(4,5-dimethylthiazol-2-yl)-2,5-diphenyl tetrazolium bromide) assay (Sigma, St. Louis, MO). Two hours before ending the 24 hour treatment, 10 µl of MTT (5 mg/ml saline) was added to each well, the samples were incubated for 2 hours at 37°C. Cells were lysed and MTT crystals solubilized by the addition of 100 µl of 0.02 N HCl in isopropanol. The absorbance of each well was determined at 590 nm using a BioTek microplate reader (BioTek instruments, Winooski, VT). Cell viability (%) was calculated relative to the control.

#### Annexin V staining

APL cell apoptosis was assessed by flow cytometry using the annexin V-FITC apoptosis detection kit as described by the manufacturers (BD Biosciences Pharmingen, San Diego, CA). Briefly, APL cells were grown in 24-well plates in the presence or absence of M2-BMSCs, fibronectin-coated plates, transwell plates, or M2-BM SN for 4 hours before treatment with chemotherapy for 24 hours. Cultures were harvested and washed once in phosphate-buffered saline (PBS) and resuspended in 1X binding buffer in PBS with 1% BSA and incubated with Annexin V-FITC and APC anti-mouse Ly-6G and Ly-6C (Gr-1) (BD Pharmingen) in the dark at room temperature for 30 minutes. Cells were then washed once with PBS and resuspended in 1X binding buffer in PBS supplemented with PI. All data was acquired on a BD FACSCanto II cytometer and analyzed using FACSDiva software (BD Biosciences, San Diego, CA). We have previously reported that APL cells can be tracked due to the co-expression of murine CD34 and the myeloid surface antigen Gr-1. [12].

#### Adherence assay

APL cells were cultured in 24-well plates alone, co-cultured with M2-BMSCs, or cultured in wells pre-coated with fibronectin for 24 hours. Then, the supernatant was carefully removed and the adhered fraction was resuspended using a cell scraper. Both fractions were stained with anti-mouse Ly-6G and Ly-6C (Gr-1) (BD Pharmingen) for 30 minutes in the dark. Samples were washed once with PBS before analysis. To quantify the relative amount of adhered cells, both cell fractions (supernatant and adhered) were acquired for 50 seconds in a BD Pharmingen FACSCanto II flow cytometer. The Gr-1^+^ events of both fractions were obtained and the percentage of adherence was determined as follows: APL adherence was calculated as the percentage of APL cells present in the adhered fraction in relation to the total amount of APL cells in both fractions.

#### Cell cycle distribution

APL cells grown in 24-well plates with or without M2-BM SN were harvested and centrifuged at 100 g for 5 minutes. Cell pellets were fixed in ice-cold 70% (v/v) ethanol in PBS overnight at 4°C by gradual mixing. Cells were subsequently stained with 200 µl/propidium iodide (PI; 10 µg/ml)/RNase (1 mg/ml) buffer (BD Biosciences Pharmingen, San Diego, CA) for 30 minutes at 37°C before flow cytometry analysis of APL cell cycle status.

#### Detection of caspase-3 activation

Caspase-3 activation was measured using the PE active caspase-3 monoclonal antibody apoptosis kit as described by the manufacturers (BD Pharmingen, San Diego, CA). Briefly, APL cells were grown in 24-well plates with or without M2-BMSCs for 2 hours before treatment with Ara-C for 24 hours. Cultures were harvested and washed twice in cold PBS. Cell pellets were resuspended in BD Cytofix/Cytoperm solution and incubated on ice for 20 minutes. Cells were then washed twice in 1X BD Perm/Wash buffer and resuspended in 1X BD Perm/Wash buffer with caspase-3 antibody for 30 minutes at room temperature. After additional washing, cells were analyzed by flow cytometry.

#### Protein extraction and western blot

Cell pellets were washed twice in ice cold PBS and lysed with lysis buffer and protease inhibitors (20 mM Tris (pH 7.5), 1% triton, 10% glycerol, 137 mM NaCl and 2 mM EDTA, 250 µM PMSF, 5 µg/ml leupeptin) for 20 min, followed by centrifugation at 10,000 rpm for 10 min at 4°C. Protein concentration of the supernatant was measured using Bio-Rad protein assay dye reagent (Bio-Rad, Hercules, CA, USA). Fifty micrograms of proteins were equally loaded to a 10% SDS-polyacrylamide gel, electrophoresed, and transferred to polyvinylidene difluoride membrane (PVDF) (Thermo Scientific, Rockford, IL, USA). Membranes were developed using Pierce® ECL Western blotting substrate (Thermo Scientific). The following antibodies were used for immunoblotting: cleaved poly (ADP-ribose) polymerase (PARP) (Cell Signaling Technology, Danvers, MA, USA), mENT1 (Abcam, Cambridge, MA, USA) and β-actin (Cell Signaling Technology).

#### Isolation of RNA and reverse transcription-polymerase chain reaction (RT-PCR)

Total RNA was extracted using Trizol (Invitrogen) as described by the manufacturer. Complementary DNA was subsequently synthesized from total cellular RNA using MMLV reverse transcriptase (Promega, Madison, WI, USA), and PCR was performed using a PCR thermal cycler (Labnet International Inc. Edison, NJ, USA). The PCR program used to amplify mENT1 and GAPDH consisted of a precycle of 5 minutes at 94°C, 30 seconds at 30°C and 30 seconds at 72°C. Following this initial cycle, the reaction was continued for 26 cycles of 30 seconds at 94°C, 30 seconds at 60°C, and 30 seconds at 72°C and concluded with 5 minutes at 72°C.The primers sequences were as follows: mENT1 forward primer: CTCATCAATT-CATTTGGTGCCA; mENT1reverseprimer: GCAAGGTTGAGCTGCAGG-TAAT; mGAPDH forward primer: CACCCAGAAGACTGTGGATGG; GAPDH reverse primer: CCACCAC-CCTGTTGCTGTAG.

#### mENT1 activity

The mENT1 activity assay was performed as described previously, [15] using a sodium free transport assay buffer (20 mM TrisHCl (pH 7.5), 3 mM K_2_HPO_4_, 1 mM MgCl_2_ 6 H_2_O, 2 mM CaCl_2_, 5 mM glucose and 130 mM N-methyl-D-glucamine (NMDG, pH 7.4). Briefly, cells were washed once with transport assay buffer and then suspended in transport assay buffer. After, pre-incubation with 1 µM NBMPR or vehicle (DMSO) for 15 minutes, uptake assays were started by adding equal volume of transport buffer containing 2 µM cold uridine, [^3^H]-uridine 4 µCi/ml plus NBMPR or DMSO. Time course of uptake under this condition was performed to determine linearity (not shown). Uptake was stopped after 5 minutes followed by five rapid washes with ice cold transport buffer containing 1 mM unlabelled uridine. The cell pellets were lysed in a lysis buffer containing 20 mM TrisHCl (pH7.5), 137 mM NaCl, 1% Triton X-100, 10% glycerol and 2 mM EDTA. After centrifugation at 1000 rpm for 10 minutes at 4°C, 80% of the supernatant was used to measure incorporated radioactivity and 20% to measure total protein content. Difference between total transport and transport in the presence of 1 µM NBMPR was defined as ENT1-mediated uridine transport.

#### Statistical analysis

All data are given as means ± S.D. of at least three independent experiments. Comparison of treatments against controls was made using one-way Analysis of variance (ANOVA) followed by Bonferronìs least significant difference post hoc test. Survival curves were generated using the method of Kaplan and Meier and analyzed by the log-rank test. Statistical analysis was performed using GraphPad Prism 5 statistical package. The significance level chosen for the statistical analysis was p<0.05.

## Results

### The BM Niche Protects Leukemia Cells from Ara-C Induced Cytotoxicity but not Radiotherapy Both in vitro and in vivo

**Figure 1:**
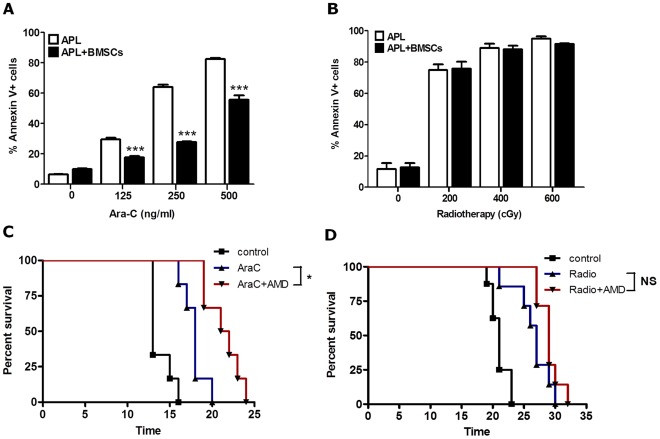
BM microenvironment modulates protection of APL cells to Ara-C induced apoptosis but not to radiation in vitro and in vivo. (A) APL cells were cultured in absence or presence of M2-BMSCs for 2 hours before treatment with Ara-C (125, 250, 500 ng/ml) or vehicle alone (control). APL cell death was assessed by flow cytometry using a GR-1-APC mouse antibody and the annexin V-FITC apoptosis kit. (B) Cultures (as described 1A) were exposed to various radiation doses (200, 400 and 600 cGy) and allowed to recover for 24 before cell death was assessed as mentioned in (1A). (C) Kaplan Meier plot of overall survival of mice. Syngeneic B6129F1 recipient mice were intravenously injected with 10^6^ APL cells. On day 12 post-APL injection, mice were left untreated (control; n = 6) or treated with AMD3100 alone (n = 7), Ara-C alone (n = 8), or the combination of AMD3100 and Ara-C (n = 8). Mice treated with chemotherapy received a single injection of Ara-C (500 mg/Kg) on days 12 and 13 post-APL injection. Mice treated with AMD3100 (5 mg/Kg) received subcutaneous injections 1 hour before and three hours after Ara-C treatment. (D) Kaplan Meier plot of overall survival of mice. Syngeneic B6129F1 recipient mice were intravenously injected with 10^6^ APL cells. On day 12 post-APL injection, mice were left untreated (control; n = 6), exposed to radiation (350 cGy) (n = 8), or the combination of AMD3100 and radiation (n = 8). Mice treated with AMD3100 (5 mg/Kg) received a single subcutaneous injection 2 hours before radiation treatment. Each bar represents the mean ± SEM of 3 independent experiments. ***p<0.001 (APL + BMSCs versus APL). Overall survival of leukemic mice is not significantly prolonged when recipients are treated with the combination of AMD3100 and radiation versus radiation cohorts).

We previously published that M2-BMSCs protect leukemic blasts from cytotoxic agents, such as Ara-C, in vitro and that disruption of the BM microenvironment in vivo with the CXCR4 antagonist AMD3100 sensitizes leukemia cells to chemotherapy. [12] In our first set of studies we repeated these prior studies with Ara-C and determined if similar results could be obtained following radiotherapy-induced apoptosis. APL cells were cultured alone or in co-culture with M2-BMSCs for 2 hours before treatment with varying doses of Ara-C or radiotherapy. Cultures were then incubated for 24 hours and cell death in the GR1+ leukemia population was measured using an annexin V-FITC apoptosis detection kit and flow cytometry. As before [12], we noted a dose-dependent increase in cell death by apoptosis in APL cells cultured in the absence of stromal cells, whereas APL cells co-cultured with BMSCs were significantly resistant to the apoptotic effects of Ara-C (Figure 1A). In contrast, co-culture of APL cells with M2-BMSCs did not provide significant protection from radiotherapy-induced apoptosis in vitro (Figure 1B). Similar studies were also performed by irradiation of APL cultures co-incubated with or without M2-BMSCs for 24 hours. Again, there was no M2-BMSC protection from radiotherapy-induced cell death (data not shown).

We next examined whether in vivo administration of AMD3100 to leukemic mice sensitized APL cells to radiotherapy similar to Ara-C. For these studies, a total of 29 healthy B6129F1 mice were injected intravenously through the tail vein with 10^6^ APL cells. Then, 12 days later, when there were approximately 3–5% blasts in the peripheral blood (data not shown), cohorts of mice were treated with AMD3100 (subcutaneous, 5 mg/Kg) and either Ara-C or radiotherapy. As we previously published, [12] treatment of leukemic mice with Ara-C alone (500 mg/kg) on days 12 and 13 after APL injection prolonged their survival compared to the untreated control (Figure 1C). Furthermore, the combination of AMD3100 (given 1 hour before and 3 hours after each Ara-C injection) and Ara-C significantly prolonged survival relative to mice treated with Ara-C alone (Figure 1C). In contrast, no survival advantage was observed following combination therapy with AMD3100 and radiotherapy (350 cGy) compared to radiotherapy alone (Figure 1D). These data indicate that disruption of the APL/BM interaction by AMD3100 sensitizes leukemia blasts to Ara-C but not radiotherapy.

### BMSCs Secrete a Soluble Factor(s) that Protects APL Cells from Ara-C Induced Apoptosis

**Figure 2:**
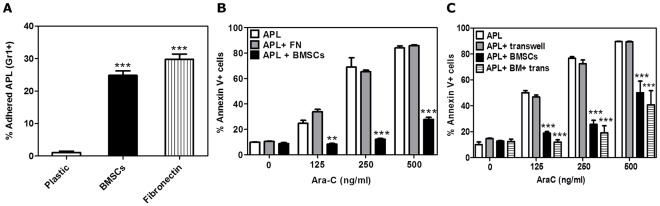
BMSCs secrete a soluble factor(s) that protects APL cells from Ara-C induced apoptosis. (A) Adherence assay: APL cells were cultured alone, co-cultured with M2-BMSCs or with fibronectin-coated plates for 24 hours. Cells were processed and quantification of APL GR1+ cells in the supernatant and adhered fractions were performed by flow cytometry analysis. Adherence was calculated as the percentage of APL cells present in the adhered fraction in relation to the total amount of APL cells in both fractions. For chemosensitivity studies: (B) APL cells were cultured alone, cultured using fibronectin pre-coated wells, or cultured with M2-BMSCs for 4 hours; (C) APL were cultured with or without M2-BMSCs or using a transwell system. Cultures were incubated for 2 hours before treatment with Ara-C (125, 250, 500 ng/ml) for 24 hours or vehicle alone (control). APL cell death was assessed by flow cytometry using the GR-1-APC mouse antibody and the annexin V-PE apoptosis kit. Each bar represents the mean ± SEM of 3 independent experiments. **p<0.01 and ***p<0.001 (all groups versus APL).

We next evaluated whether physical interaction of BMSCs and APL cells was necessary for chemoprotection. APL cells were cultured alone (plastic), co-cultured with M2-BMSCs, or cultured in fibronectin coated wells. The apoptosis assays were performed by flow cytometric quantification of GR1+ cells in the supernatant and adhered fractions co-cultured for 24 hours. Adherence was calculated as the percentage of APL cells present in the BMSC-bound fraction in relation to the total amount of APL cells in both fractions. As observed in figure 2A, there is a significant physical cell interaction between APL cells with the M2-BMSCs, and fibronectin.

We next investigated whether the physical interaction of APL with fibronectin resulted in chemoprotection of APL cells to Ara-C-induced apoptosis. APL cells were cultured with or without fibronectin-coated wells or co-cultured with M2-BMSCs. Cultures were treated with Ara-C for 24 hours and cell death was measured by flow cytometry. As demonstrated in Figure 2B, fibronectin failed to protect APL cells against Ara-C-induced apoptosis while APL cells bound to M2-BMSCs were resistant to Ara-C.

**Figure 3:**
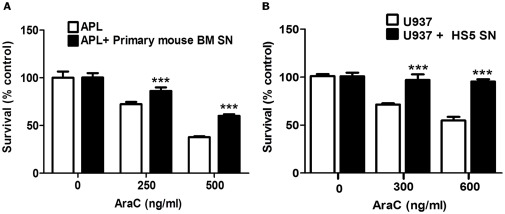
Primary mouse and human BMSCs supernatant protect leukemia cells from Ara-C induced cytotoxicity. APL and U-937cells were cultured with or without primary mouse BM stromal cell supernatant (PM-BM SN) or human BM SN (HS5-BM SN) for 2 hours before treatment with Ara-C (0, 250 and 500 ng/ml) (A) or Ara-C (0, 300 and 600 ng/ml) (B) for 24 hours. Leukemia cell viability was assessed by the MTT assay. Each bar represents the mean ± SD of 3 independent experiments. ***p<0.001 (leukemia cells versus leukemia cells + mouse or human BM SN).

To test whether the supernatant from M2-BMSCs could modify the chemosensitivity of the APL cells, we performed transwell assays. APL cells were cultured alone, cultured in upper transwell chambers (as a control to confirm optimum access of the drug to the upper chamber), co-cultured with M2-BMSCs or co-cultured with BMSCs using transwell chambers. Cultures were treated with Ara-C for 24 hours and apoptosis of APL cells was analyzed as previously described. As shown in Figure 2C, we observed similar chemoprotection following culture of APL cells in the upper chamber of a transwell compared to cells on direct contact with M2-BMSCs. These results suggest that M2-BMSCs secrete a soluble factor(s) that protects APL cells from Ara-C-induced apoptosis. Furthermore, this chemoprotection was also observed in APL cells cultured with primary mouse BM supernatant (PM-BM SN) (Figure 3A) and human leukemia cells (U-937 cell line) cultured with human BM supernatant (HS5-BM SN) (Figure 3B).

### M2-BMSCs Protect APL Cells from Ara-C Induced Caspase-3 Activation and PARP Cleavage

**Figure 4:**
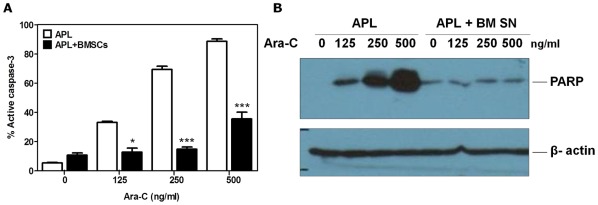
BMSCs mediated chemoprotection prevents the activation of the apoptotic cell death pathway. APL cells were cultured alone or co-cultured with M2-BMSCs for 2 hours before treatment with Ara-C (125, 250 and 500 ng/ml) or vehicle alone (control) for 24 hours. (A) Caspase-3 activation was assessed by flow cytometry using a PE-active caspase-3 monoclonal antibody and mouse GR-1-FITC antibody. (B) Cell lysates were processed for western blot analysis to detect cleaved PARP using a mouse monoclonal antibody. Each bar represents the mean ± SD of triplicates from individual experiments. *p<0.05; and ***p<0.001 (APL versus APL + BMSCs).

To confirm the results obtained with Annexin V staining and to mechanistically study the pathways involved in Ara-C-induced cytotoxicity, we measured the activation of caspase-3 and PARP cleavage to confirm activation of the apoptotic cell death pathway. Figure 4A demonstrates a dose–dependent increase in caspase-3 activation following treatment of APL cells with increasing concentrations of Ara-C in the absence of M2-BMSCs. In contrast, co-culture of APL cells with M2-BMSCs resulted in a significant reduction in caspase-3 activation in the presence of Ara-C. Similarly, M2-BM SN had a potent inhibitory effect on Ara-C induced PARP cleavage in the APL cells (Figure 4B). These data further confirm that M2-BMSCs and soluble factors released by M2-BMSCs protect APL from Ara-C-induced apoptosis.

### M2-BM SN does not Alter APL Cell Cycle Progression or Result in Cell Cycle Arrest

**Figure 5:**
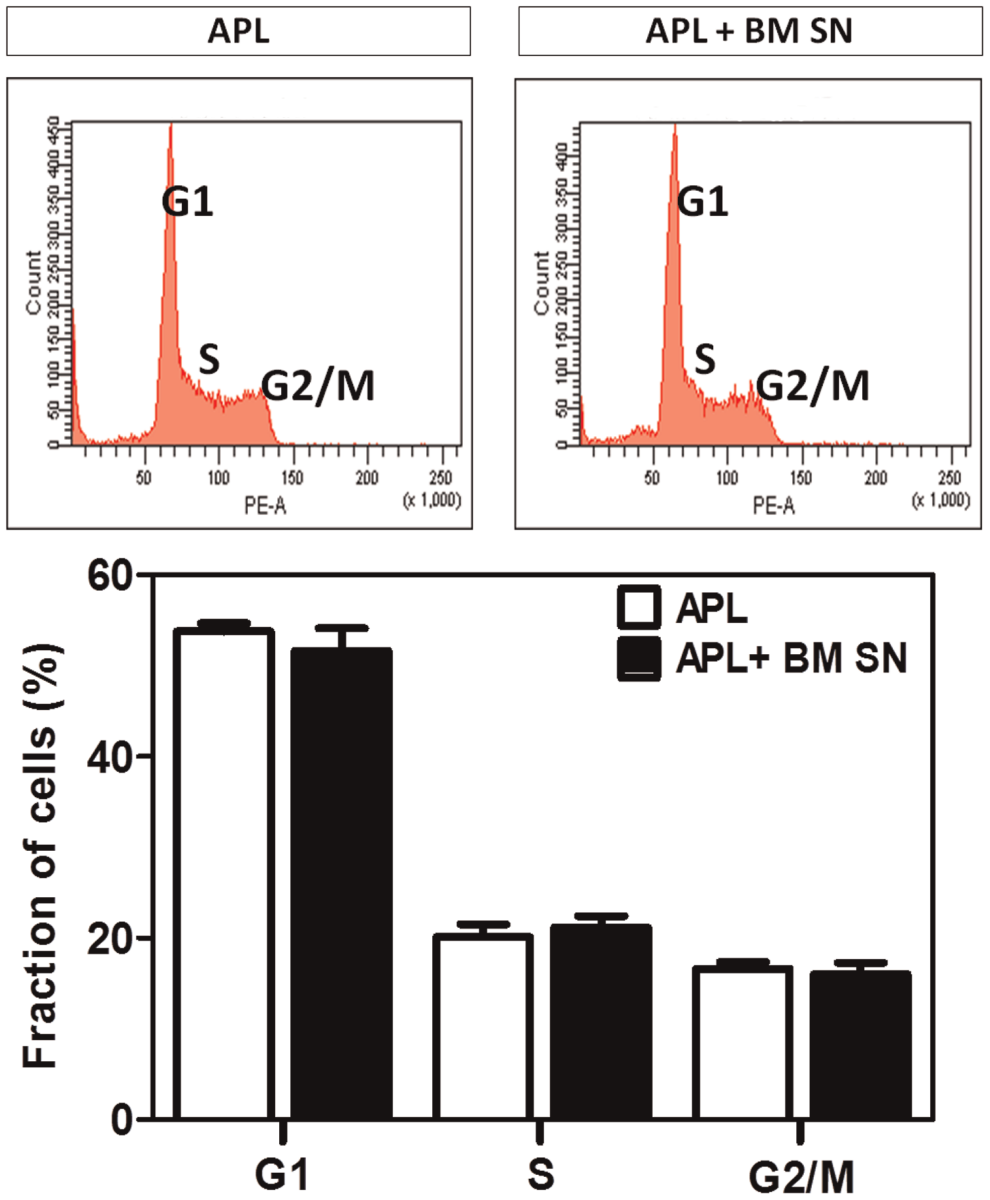
BMSCs supernatant caused no APL cell cycle arrest. APL cells were cultured with or without M2-BM SN for 24 hours. Cells were harvested, fixed and stained with PI and analyzed by flow cytometry. Upper panels show representative histograms of APL cells in the absence (left upper panel) and presence of M2-BM SN (right upper panel). Lower figure shows mean and SD of three separate experiments.

To verify whether BMSC induced chemoprotection against Ara-C was associated with cell cycle alterations or cell cycle arrest, APL cells were cultured with or without M2-BM SN, stained with PI and analysed by flow cytometry. Figure 5 shows representative cell cycle histograms of the APL cells and the quantification of the relative number of APL in each stage of the cell cycle (G1, S, G2/M). These data demonstrate that the M2-BM SN is not associated with APL alterations of cell cycle or accumulation of APL in G1 (cell cycle arrest).

### Selective in vitro Sensitivity of APL Cells to Other Chemotherapeutic Agents

**Figure 6:**
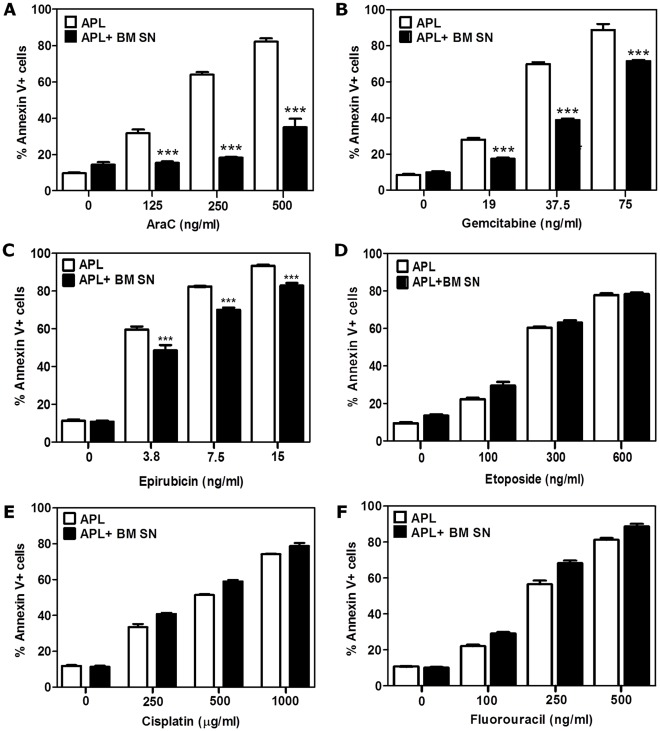
In vitro sensitivity of APL cells to various chemotherapeutic agents. For chemosensitivity studies: APL cells were cultured alone or with M2-BM SN for 2 hours before treatment with Ara-C (125, 250 and 500 ng/ml) (A), gemcitabine (50, 100 and 200 ng/ml) (B), epirubicin (3.8, 7.5, 15 ng/ml) (C), etoposide (150, 300 and 600 ng/ml) (D), cisplatin (250, 500, 1000 µg/ml) (E), 5-fluorouracil (100, 250 or 500 ng/ml) (F), or vehicle alone (control) for 24 hours. APL apoptosis was assessed by flow cytometry with annnexin V-PE apoptosis kit. Each bar represents the mean ± SD of 3 independent experiments. ***p<0.001 (APL versus APL + BM SN).

To further understand whether the BMSC-mediated chemoprotection to Ara-C-induced apoptosis also occurred with other cytotoxic agents, we performed several chemosensitivity studies with various anti-metabolites widely used in the treatment of cancer. These include the pyrimidine analogues (Ara-C and gemcitabine), anti-folates (5-Fluorouracil), anthracyclines (epirubicin), topoisomerase inhibitors (etoposide), and platinum-based agents (cisplatin). APL cells were cultured with or without M2-BM SN and treated with each cytotoxic agent for 24 hours before analysis of cell death by flow cytometry using the annexin V-FITC apoptosis detection kit (Figure 6). As observed in Figure 6, the M2-BM SN significantly reduced apoptosis of APL cells after Ara-C (Figure 6A), gemcitabine (Figure 6B) and epirubicin (Figure 6C) treatment but not after exposure of APL cells to etoposide, cisplatin or 5-Fluorouracil (Figures 6 D-F). These data suggest that, in vitro, M2-BMSCs provide APL cells with selective chemoresistance against specific nucleoside analogues (Ara-C and gemcitabine) and anthracyclines (epirubicin) but not to cisplatin, 5-Fluorouracil or etoposide.

### M2-BM SN Modulates mENT1 Activity to Protect APL Cells from Ara-C Induced Apoptosis

**Figure 7:**
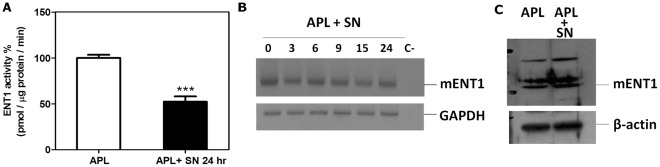
BMSC soluble factor(s) inhibits mENT1 activity and protect APL cells from AraC-induced apoptosis. (A) Measurement of mENT1 activity. APL cells were cultured alone or with M2-BM SN for 24 hours. Cells were harvested and mENT1 activity was measured by the incorporation of radioactive ^3^H-adenosine (B) RNA extraction from APL cells cultured alone or in presence of M2-BM SN for a time-course of 0, 3, 6, 9, 15, and 24 hours was used for RT-PCR to detect expression of mENT1. (C) Western blot analysis for mENT1 using APL cells with or without M2-BM SN for 24 hours. Each bar represents the mean ± SD of 3 independent experiments. ***p<0.001 (APL versus APL SN).

Transport of Ara-C across the cell membrane is primarily dependent on ENT-1. We therefore tested whether the M2-BMSC induced chemoprotection to Ara-C involved alterations of mENT-1 activity. To measure mENT1 activity, APL cells were cultured with or without M2-BM SN for 24 hours and mENT1 activity was quantitated by measuring transport and incorporation of ^3^H-adenosine into APL cells. Figure 7A shows that the addition of M2-BM SN significantly decreased mENT1 activity 50% compared to APL cells incubated in fresh medium. Importantly, we found no change in the expression of mENT1 at the mRNA (Figure 7B) or protein (Figure 7C) level in APL cells cultured with M2-BM SN. These data suggest that soluble factor(s) derived from M2-BMSCs modulate mENT1 activity which is not associated with any change of either mRNA or proteins levels of mENT1.

**Figure 8:**
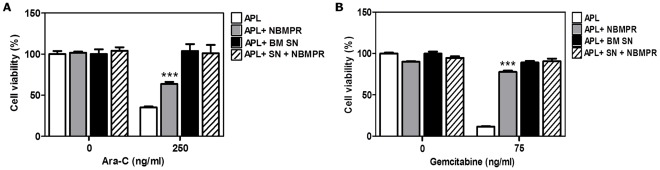
mENT1 inhibition by NBMPR chemoprotects APL cells from Ara-C and gemcitabine-induced cell death. APL cells were cultured alone or with M2-BM SN with pre-treatment with NBMPR (1 µM) for 2 hours before normal treatment with Ara-C (250 ng/ml) (A) or gemcitabine (75 ng/ml) (B) for 24 hours. Cell viability was measured by the MTT assay. Data are the mean ±SD of at least three independent experiments. ***p<0.001 (APL versus APL + NBMPR).

To implicate mENT1 as one of the pathways for Ara-C chemoprotection seen when APL cells are incubated with M2-BM SN, APL cells were incubated with M2-BM SN in the presence or absence of a selective mENT1 small molecule inhibitor, NBMPR. NBMPR (1µM) was added 2 hours before treatment with Ara-C or gemcitabine for 24 hours. Cell viability was measured by the MTT assay. As observed in Figure 8 A–B, the inhibition of mENT1 transporter by NBMPR conferred a significant chemoprotection to both cytotoxic agents when administered in the absence of M2-BM SN. These data suggest that soluble factor(s) derived from M2-BMSCs inhibit mENT1 nucleoside transporter activity resulting in resistance of APL cells to the apoptotic effects of both Ara-C and gemcitabine and that this chemoprotective effect can be phenocopied by small molecule inhibitors of mENT1.

## Discussion

Novel strategies to improve outcomes in AML are urgently needed especially for those patients who fail remission-induction or who relapse where chemotherapy resistance is likely to play a major role in limiting overall and leukemia free survival. [16] The importance of tumor microenvironment for cancer progression and in particular the interaction of leukemia cells with the BM stroma are increasingly being recognized as a critical factor in mediating cancer development and drug resistance. [17] In this study, we show that BMSCs secrete a soluble factor(s) that specifically protects leukemic cells from Ara-C-induced apoptosis and inhibits mENT1 activity in an in vitro leukemia model.

The interaction between HPC and the BM niche is mediated by several adhesion molecules expressed on the HPC surface with their cognate receptors in the BM stroma including CXCR4/stromal cell–derived factor-1 (SDF-1 or CXCL12). [18], [19] CXCR4 is also constitutively expressed in several hematopoietic malignancies. [20], [21] The CXCR4/SDF-1 axis regulates the trafficking of HPC to and from the BM, [22] and mediates tumor cell homing to BM in both hematopoietic [23] and solid tumors. [24], [25] Others [26] and we [12] have previously shown that AMD3100 sensitizes AML cells to the cytotoxic effects of chemotherapy presumably through disruption of the protective BM microenvironmental niches. In this study, we failed to observe a similar sensitization effect following treatment of leukemic mice with AMD3100 and radiotherapy. Furthermore, although our in vitro studies demonstrated that M2-BMSCs provide chemoprotective effects, they fail to inhibit the apoptosis induced by radiotherapy. Taken together, these data suggest that the BM stroma environment provides protection against chemotherapy but not radiotherapy.

The interaction between leukemic cells and stromal fibronectin is a decisive factor for minimal residual disease of AML. This interaction is mediated by VLA-4 receptor. [27] Patients whose AML blasts express low surface VLA-4 had better response to chemotherapy and improved survival suggesting that the VLA-4 pathway may also play a role in mediating resistance to chemotherapy similar to the CXCR4/SDF-1 axis. [28] Our in vitro data, however, suggest that binding of APL cells to plate bound fibronectin had no impact on the sensitivity of APL cells.

To further define the mechanisms involved in leukemia chemoprotection by BMSCs, we focused our research on Ara-C mediated cytotoxicity. We first demonstrated that the co-culture of APL with M2-BMSC cells in vitro resulted in enhanced survival after Ara-C chemotherapy compared to APL cells without BMSCs. In addition to cell-cell contact, recent reports have shown that BMSCs in vivo produce a cocktail of growth factors and cytokines that enhance leukemia growth and progression through autocrine and paracrine effects with a bidirectional crosstalk between leukemia blasts and BM microenvironment. [29], [30] We showed that APL cells co-cultured with M2-BM SN, PM-BM SN, or M2-BMSCs using transwell assays were significantly protected from Ara-C-induced apoptosis. This data indicates that the M2-BMSCs and primary mouse BMSCs secrete a soluble factor(s) that protects APL cells from Ara-C mediated apoptosis. To confirm that this chemoresistance was not unique to APL cells, we also showed that the human AML cell line (U-937 ) was protected from Ara-C cytotoxicity following culture with HS5-BM SN. Furthermore, to determine if the cytoprotective effect of M2-BMSCs was unique to Ara-C, we tested the effects of other cytotoxic agents including, epirubicin, 5-FU, cisplatin and gemcitabine. While we acknowledge that some of these agents (5-FU and cisplatin) are not used clinically in the treatment of AML, they all induced apoptosis of APL cells in a dose-dependent manner. Therefore, we sought to determine if BMSCs provided protection against the cytotoxic effects of these different drug classes similarly to Ara-C. Interestingly, although M2-BMSCs provided slight protection against epirubicin induced apoptosis, none of the other treatments (with the exception of gemcitabine, a nucleoside analogue related to Ara-C) had any effects in vitro. Taken together, this data suggests that, at least in vitro, BMSCs primarily provide protection against the cytotoxic effects of pyrimidine analogues and anthracyclines, the two most common drug classes used in the treatment of AML.

The maintenance of leukemia precursors in a quiescent state, may favor survival when exposed to cell cycle dependent cytotoxic agents such as Ara-C. To determine if M2-BM SN induced cell cycle arrest, we performed cell cycle analyses of APL cells in the presence or absence of M2-BM SN. We found no significant changes in the cell cycle status of APL cells cultured inM2-BM SN.

Three mechanisms have been proposed for Ara-C resistance. [31] First, Ara-C cytotoxicity depends strongly on its intracellular conversion to the 5-triphosphate form, Ara-CTP. Ara-C is first phosphorylated by deoxycytidine kinase (dCK), which is then converted to Ara-CTP by other cellular kinases. [32] Alterations in expression or activity of the *dCK* gene correlated with clinical outcome in Ara-C treated AML patients. [33], [34] The second resistance mechanism is associated with increased levels of the catabolic enzymes such as cytidine deaminase or 5-nucleotidases and increased levels of expression of DNA polymerases. [35] Finally, prior reports suggested that a deficiency of hENT1 confers high-level resistance to Ara-C in leukemia cells. [10], [11], [36] hENT1 is responsible for up to 80% of Ara-C influx into human leukemic cells. [37], [38] Previous reports showed that a lower expression of *hENT1* mRNA was related to a shorter disease-free survival in patients with AML. [36] In addition, elevated *hENT1* mRNA expression explained the remarkable Ara-C sensitivity of infants with *MLL* gene-rearranged ALL. [39] In pancreatic cancer, patients treated with gemcitabine with high hENT1 expression were associated with better outcome. [39] One of the most frequent class I mutations in AML is fms-related tyrosine kinase 3-internal tandem duplications (FLT3-ITD), which occur in about 25% of AML patients. Interestingly, a recent report suggested that the FLT3-ITD specifically induces Ara-C resistance in AML through the repression of ENT1 expression [40].

To determine the mechanisms by which BMSCs protect APL cells from Ara-C induced apoptosis, we explored the mENT1 activity of the APL cells cultured with and without M2-BM SN. We showed that M2-BMSC secreted soluble factor(s) caused a significant reduction in mENT1 activity. We then found no change in the expression of mENT1 at the RNA and protein levels. We further examined the effect of mENT1 transporter inhibitor, NBMPR on Ara-C and gemcitabine induced apoptosis of APL cells. Our data show that NBMPR-mediated inhibition of mENT1 transporter significantly protected APL to the apoptotic effects of both Ara-C and gemcitabine similar to M2-BM SN. Taken together, our data suggest that the M2-BMSC secreted soluble factor(s) inhibit mENT1 activity, thereby potentially reducing Ara-C and gemcitabine uptake into the APL cells resulting in reduced apoptosis. In addition, ENT1 is not resposible for the celular uptake of anthracyclines such as epirubicin. Their cellular incorporation occurs via the multidrug resistance protein (MDR) family of transport proteins. [41], [42] Therefore, the soluble factor(s) secreted by the BMSCs must modulate other cellular pathways that contribute to the resistance of APL cells to anthracycline-induced apoptosis.

In summary, our data suggest that the BMSCs protect APL cells from Ara-C-induced apoptosis both in vitro and in vivo and that this effect is mediated, in part, by soluble factor(s) released by the stromal cells. This effect does not require direct contact between leukemic cells and BMSCs in vitro. Furthermore, the soluble factor(s) inhibits leukemic cell mENT1 transporter activity which may result in selective resistance by nucleoside-based chemotherapy agents that are transported by mENT1. There is substantial evidence suggesting that leukemic cell interaction with the BM niche is essential for leukemia survival, resistance to treatment and disease progression. To our knowledge, this is the first report describing modulation of mENT1 activity by a soluble factor secreted from BMSCs. Approaches to target the niche like disrupting the cross talk between leukemia cells and their microenvironment remains a promising approach for treating hematological malignancies, and may potentially modulate leukemia response to conventional chemotherapy and improve outcomes.
